# Predicting early recurrence of colorectal cancer liver metastases: an integrative approach using radiomics and machine learning

**DOI:** 10.3389/fonc.2025.1613093

**Published:** 2025-11-14

**Authors:** Yanzong Lin, Yunxia Huang, Zhaohui Liu, Xiaobin Feng, Chunkang Yang

**Affiliations:** 1 Department of Colorectal Surgery, Clinical Oncology School of Fujian Medical University, Fujian Cancer Hospital, Fuzhou, China; 2 Department of General Surgery, First Affiliated Hospital of Xiamen University, Xiamen, Fujian, China; 3 Department of Radiation Oncology, First Affiliated Hospital of Xiamen University, Xiamen, Fujian, China; 4 Hepatopancreatobiliary Center, Beijing Tsinghua Changgung Hospital, Institute for Precision Medicine, Key Laboratory of Digital Intelligence Hepatology (Ministry of Education), Tsinghua University, Beijing, China

**Keywords:** colorectal liver metastasis, CT radiomics, machine learning, intrahepatic recurrence, prediction

## Abstract

**Background:**

The overall incidence of liver metastasis in colorectal cancer is as high as 50%, and surgery remains the only potentially curative approach for the metastatic disease. The recurrence rate of liver metastases within one year after surgery is still 60%-70% in clinical practice. Whether we can accurately predict the early recurrence of patients after surgery is one of the most important considerations in formulating the overall treatment strategy.

**Methods:**

In this study, we combined radiomics feature extraction with machine learning classification methods to develop a novel strategy for predicting intrahepatic metastases based on imaging radiomics and machine learning. We constructed and systematically evaluated multiple machine learning models to assess their performance. By validating these models on a test set, we determined the effectiveness of each predictive model and selected the one with the highest predictive accuracy.

**Results:**

The integration of radiomics and machine learning methods demonstrated significant potential in predicting intrahepatic recurrence within one year after surgery in patients with colorectal cancer liver metastases. The Gradient Boosting, LightGBM, and Random Forest models all achieved classification accuracies (ACC) exceeding 65% across all classification tasks. Notably, the Random Forest model exhibited the best performance; while its classification accuracy was 65.52% in the imaging-only group, it increased to 75.86% when both imaging and clinical information were combined, with an area under the receiver operating characteristic curve (AUC) of 70.83%, indicating strong predictive capability. These findings suggest that these models have potential application value in supporting the diagnostic work of clinical radiologists, potentially helping to reduce workload and decrease the risk of misdiagnosis.

**Conclusions:**

The imaging omics model and the combined model have good predictive efficacy for the recurrence of colorectal cancer liver metastases within one year, and can be used to assist in the clinical stratification of postoperative patients and identify high-risk factors for poor prognosis.

## 1 Introduction

Colorectal cancer (CRC) ranks as the third most frequently diagnosed cancer, with more than 1.92 million newly diagnosed cases and 903,800 deaths worldwide in 2022 (1), and its high mortality rate is mainly attributed to metastasis (2, 3). The liver is the most common site of metastasis, accounting for approximately 50% of colorectal cancer metastases (4). Liver metastasis is one of the major causes of death for CRC with 6.9 months of median survival receiving only palliative care (5, 6). Despite decades of advances in systemic and local therapies, hepatectomy remains the only curative treatment for patients with resectable colorectal liver metastases (7, 8). However, around 30% patients experience intrahepatic recurrence within a year of post-hepatectomy (9), which have worse outcomes than that late recurrence (10, 11). Therefore, accurate predictive models for early recurrence risk in patients with colorectal liver metastases are of significant clinical value.

With the advance rise of artificial intelligence technology, there are more and more researches on the application of imaging omics and machine learning to predict the prognosis of colorectal cancer liver metastasis, especially recurrence (12–14). Specifically, Lam CSN et al. employed a machine learning model for colorectal liver metastasis post-hepatectomy prognostications, presenting a better prediction ability compared to Fong Clinical Risk Score (12). Mühlberg A et al. established a imaging-based prediction model for 1-year survival of colorectal liver metastasis patients, which showed a better discriminative performance than clinical models (15).

**Figure 1:**
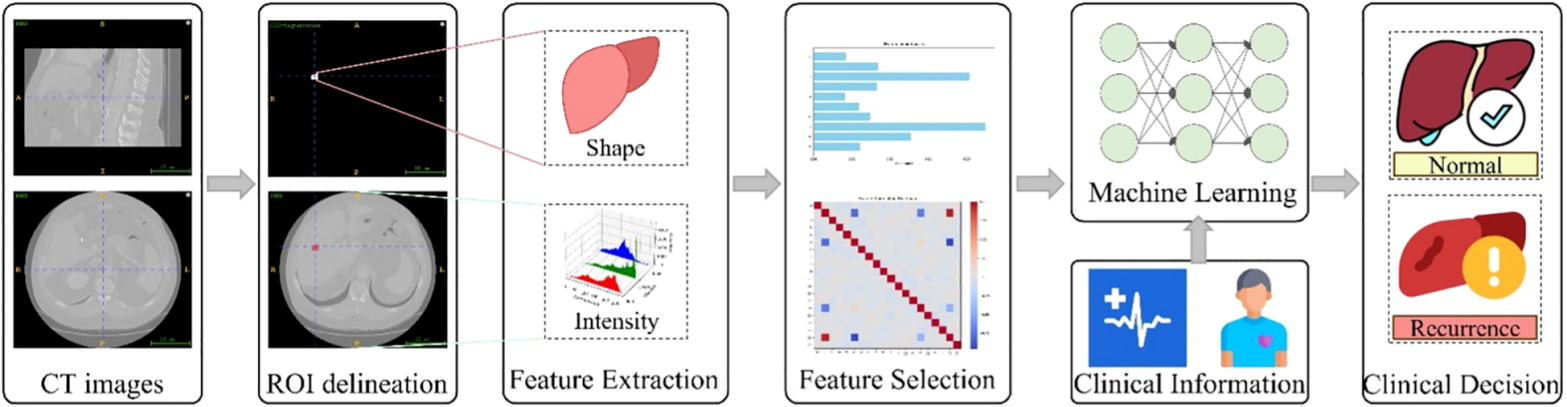
The workflow of this study.

This study aimed to develop and validate a predictive model that integrates radiomics features with machine learning algorithms to forecast intrahepatic recurrence within one year after surgery in patients with colorectal cancer liver metastases. We utilized CT radiomics data combined with an expert-annotated patient dataset, extracting radiomics features from tumor regions delineated by specialists on CT images. Two models were constructed: one using only radiomics features, and another that combines radiomics features with clinical information. Fifteen machine learning algorithms were employed for feature recognition and classification, and the most effective model was identified through performance comparison. This approach aims to predict the likelihood of recurrence in patients using radiomics technology. The workflow of this study is illustrated in **Figure 1**.

## 2 Method

### 2.1 Data acquisition

#### 2.1.1 Ethical approval

All research processes were conducted in accordance with Helsinki Declaration (revised in 2013). This study was approved by the Institutional Review Committee of the Clinical Oncology School of Fujian Medical University.

#### 2.1.2 Data source and variables

The data utilized in the experiment is sourced from the Cancer Imaging Archive dataset(TCIA). TCIA is a public database dedicated to cancer research, encompassing medical imaging and clinical data of various cases. Patients were excluded for the following:(I) liver metastasis assessment unresectable,(II) patients who cannot tolerate surgery or died perioperatively, and (III) extrahepatic recurrence. Collect the basic information of the patient, including age, gender, major comorbidity, body mass index, regional lymph node, multiple metastase, carcinoembryonic antigen(CEA) levels, max tumor size, lobar involvement and preoperative portal vein embolization. This study included a total of 197 patients with colorectal cancer liver metastasis after surgery, including 117 males (59.4%) and 80 females (40.6%); 110 patients (55.8%) were aged ≥60 years; 41 patients (20.8%) had liver metastatic tumors with a diameter of ≥5 cm; 122 patients (61.9%) received systemic chemotherapy before surgery; and 23 patients (11.7%) received portal vein embolization therapy before surgery. During the follow-up period, 122 patients experienced intrahepatic recurrence, and 37 patients experienced intrahepatic recurrence within one year after surgery (**Table 1**).

**Table 1:** Demographic and tumor characteristics of patients with colorectal liver metastasis.

Variables	Total,n (%) (n=197)	Early intrahepatic recurrence		*P*
		Yes	No	
Gender				0.135
Male	117 (59.4)	26	91	
Female	80 (40.6)	11	69	
Age				0.808
<60	87 (44.2)	17	70	
≥60	110 (55.8)	20	90	
BMI (kg/m^2^)				0.129
<24	55 (27.9)	15	40	
24-28	62 (31.5)	8	54	
>28	80 (40.6)	14	66	
Major comorbidity				0.203
Yes	109 (55.3)	17	92	
No	88 (44.7)	20	68	
Regional lymph node				0.691
Positive	69 (35.0)	14	55	
Negative	128 (65.0)	23	105	
synchronous_crlm				0.429
Yes	111 (56.3)	23	88	
No	86 (43.7)	14	72	
multiple metastases				0.339
Yes	114 (57.9)	24	90	
No	83 (42.1)	13	70	
clinrisk stratified				0.527
Yes	51 (25.9)	12	39	
No	117 (59.4)	21	96	
unknown	29 (14.7)	4	25	
CEA (ng/mL)				0.702
<5	66 (33.5)	12	54	
≥5	102 (51.8)	21	81	
unknown	29 (14.7)	4	25	
Max tumor size (cm)				0.301
<5	156 (79.2)	27	129	
≥5	41 (20.8)	10	31	
Lobar involvement				0.075
Yes	86 (43.7)	21	65	
No	111 (56.3)	16	95	
Preoperative chemotherapy				0.008
Yes	122 (61.9)	30	92	
No	75 (38.1)	7	68	
Preoperative portal vein embolization				0.128
Yes	23 (11.7)	7	16	
No	174 (88.3)	30	144	
NASH				0.413
Yes	70 (35.5)	11	59	
No	127 (64.5)	26	101	

CEA, Carcinoembryonic antigen.

#### 2.1.3 Follow-up criteria

The day of liver metastasis surgery is used as the starting point for follow-up, and follow-up is conducted by means of follow-up visits and telephone follow-up. Early intrahepatic recurrence is defined as intrahepatic recurrence within one year after resection of liver metastases, and no extra-hepatic recurrence. Patients with intrahepatic recurrence within one year after surgery are included in the recurrence group. Patients without intrahepatic recurrence within one year after surgery are included in the non-recurrence group. Follow-up is conducted every 3 months after surgery, including serum markers, abdominal B-ultrasound, abdominal CT, magnetic resonance imaging of the liver, and colonoscopy.

### 2.2 Data preprocessing

Prior to feature extraction, this study systematically preprocessed the acquired CT images using the SimpleITK package in Python. Histogram equalization was applied as a technique aimed at enhancing the contrast of images, making the details more pronounced and clear. By adjusting the grayscale distribution, histogram equalization enhances details in low contrast areas, thereby improving the visibility and analytical quality of the images. Subsequently, the images were processed using a Gaussian high-pass filter. This filter eliminates low-frequency noise, enhancing the visibility of edges and texture features within the image, which are crucial for extracting boundary information. “low-frequency noise” refers to areas in the image where the background grayscale changes slowly, such as large areas of uniform blur, brightness drift, or artifacts, which often obscure high-frequency details such as edges and textures. These boundary details were reintegrated into the original image to further enhance the representation of texture features. These preprocessing steps significantly enhanced the expression of detail information in CT images, thereby effectively improving the model’s performance.

Furthermore, to address the issue of class imbalance in the dataset, this study implemented data augmentation. Synthetic Minority Over-sampling Technique (SMOTE) was used to enhance the training data by generating new samples to balance the number of samples across different classes. This process effectively mitigated the challenges posed by class imbalance, enhancing the model’s generalization ability and predictive performance. SMOTE in this study is limited to the radiomics feature space. Specifically, after delineating and extracting features from CT image ROIs, the extracted structural features are oversampled, rather than synthesizing new images at the original image or texture level.

### 2.3 Manual region of interest annotation

**Figure 2:**
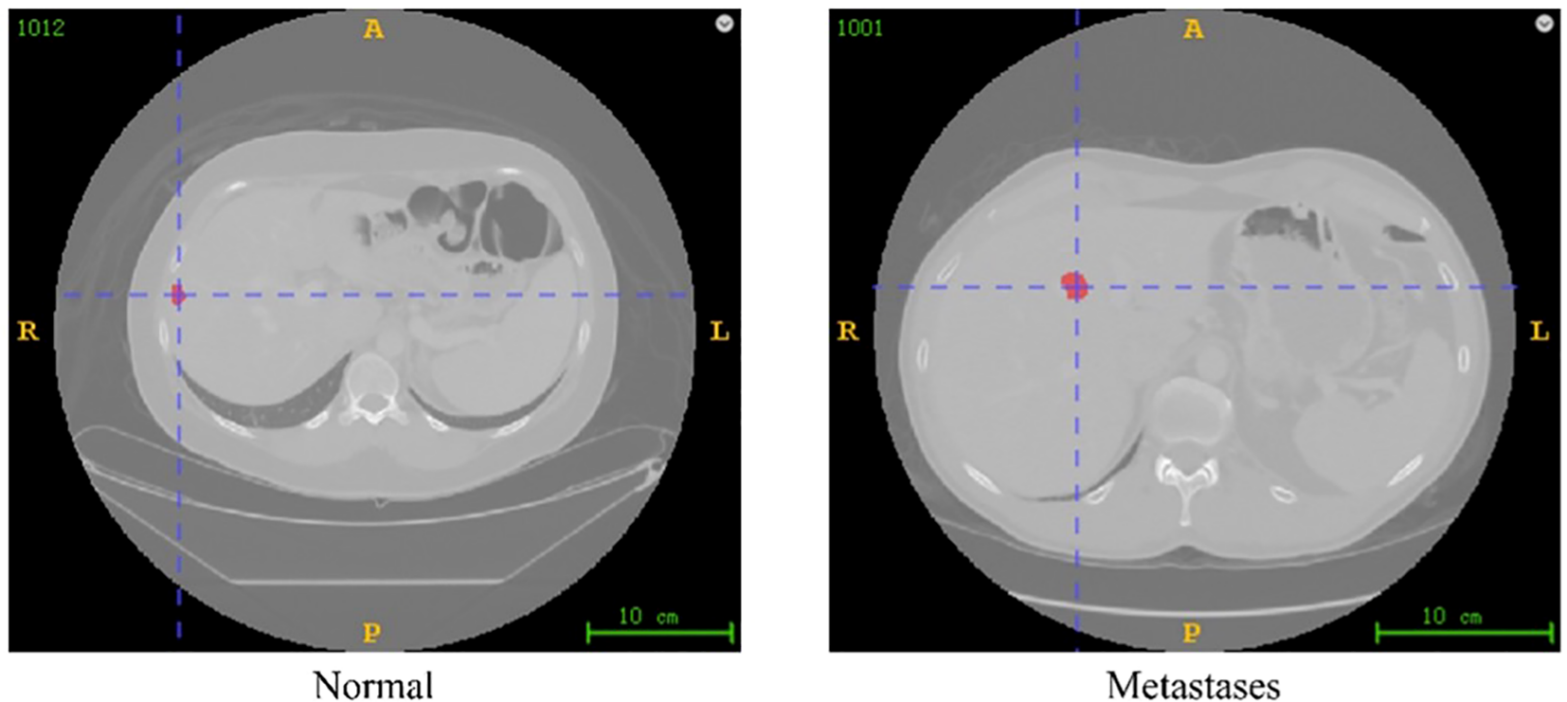
The schematic of colorectal-liver-metastases database.

Images of colorectal cancer liver metastases were exported from the TCIA database and imported into ITK⁃SNAP software in DICOM format. Regions of interest for lesions on the images were manually delineated layer by layer by two radiologists with 5–10 years’ experience in abdominal imaging diagnosis. In case of differing opinions, the decision was made by a radiology department deputy chief physician with over 15 years’ experience. All physicians involved in the delineation were unaware of the patient’s prognosis (**Figure 2**).

### 2.4 Feature extraction and selection

In this study, the feature extraction process utilized the pyradiomics library in Python, an advanced open-source tool designed to extract comprehensive radiomic features from two-dimensional (2D) and three-dimensional (3D) medical imaging data, encompassing aspects such as shape, intensity, and texture (16). Given the large number of radiomic features directly extracted by the pyradiomics library, a series of feature selection steps were implemented to optimize the feature set. Initially, features outside the 95% confidence interval were excluded via T-tests to reduce dimensionality and noise, thereby decreasing the computational burden in subsequent feature selection stages. This approach not only enabled the feature selection model to more effectively identify features that significantly contribute to the predictive target but also enhanced the model’s performance and predictive accuracy. Subsequent to this initial reduction, lasso regression was employed for further feature selection. This autoregressive technique filtered out significantly contributive features, effectively preventing overfitting and enhancing the interpretability and performance of subsequent machine learning models. Additionally, a combined clinical+radiomic group was established by integrating postoperative one-year clinical information of patients with colorectal cancer liver metastases with radiomic features. This approach was designed to utilize a more extensive set of data for model building, combining a wide range of features to facilitate personalized and precision medicine, ultimately aiming to improve model performance.

### 2.5 Construction of machine learning models

This research employed fifteen machine learning models—Random Forest (RF), Light GBM, Gradient Boosting, K-Nearest Neighbors (KNN), Decision Tree, AdaBoost, Extra Trees, Latent Dirichlet Allocation (LDA), Bootstrap Aggregating (Bagging), Bernoulli Naive Bayes (Bernoulli NB), Calibrated Classifier, Gaussian Naive Bayes (GNB), Logistic Regression, Multilayer Perceptron (MLP), and Quadratic Discriminant Analysis (QDA)—to process and classify selected radiomics features for predicting intrahepatic recurrence within one year post-surgery in patients with colorectal cancer liver metastases.

The Random Forest model leverages ensemble learning techniques, incorporating randomness during the training phase by training each tree on slightly different subsets of data and selecting the best features from randomly chosen subsets at split nodes. This significantly enhances model diversity, reduces overfitting risks, and improves generalization capabilities (17). Light GBM, an efficient gradient boosting framework, optimizes the speed and memory efficiency of traditional GBDT using a histogram-based decision tree algorithm, while maintaining high accuracy. Gradient Boosting models iteratively train decision trees to minimize loss functions, with each tree learning from the prediction residuals of the previous tree, thereby reducing model bias and enhancing accuracy on training data (18).

The KNN algorithm, a basic yet widely used method for classification and regression, predicts the value of unknown data points by referencing information from the nearest samples. In classification tasks, the category of a sample is determined by one or more of its nearest neighbors (19). Decision Tree, a non-parametric supervised learning method applicable for classification and regression tasks, predicts target values by learning decision rules from features, offering excellent interpretability due to its ability to visualize and simulate human decision-making processes (20).

AdaBoost is an ensemble learning algorithm that aims to build a strong learner by combining multiple weak learners. It reweights training samples in each round to increase the weights of previously misclassified samples, thus focusing subsequent learners on more challenging samples (21). Extra Trees, a variant of Random Forest, introduces additional randomness by selecting thresholds randomly for each feature rather than calculating the optimal thresholds, thereby enhancing tree diversity and reducing both model variance and, typically, computation time (22).

Although LDA is inherently an unsupervised learning model primarily used for identifying topic distributions in document collections, it can also be indirectly applied to classification tasks by transforming documents into topic-based representations and using these topic distributions as features for classifier training (23). Bootstrap Aggregating is a classic ensemble learning method that combines multiple models to enhance the accuracy of predictions and reduce model variance, using multiple training subsets to stabilize and improve the accuracy of final predictions (24).

BernoulliNB is a specific type of Naive Bayes classifier, designed for binary feature classification tasks. It performs well in text data handling, such as document classification or other binary classification problems, by assuming conditional independence of features within each class, simplifying computation. The Calibrated Classifier is an adjusted classifier that refines prediction probabilities to more accurately reflect the likelihood of actual events, providing more reliable probability estimates that enhance decision-making and risk assessment (25).

GNB is a classification algorithm based on Naive Bayes theory, particularly suitable for scenarios where features adhere to a Gaussian (normal) distribution. It excels in medical predictions and other areas, effectively handling continuous data and providing classification results based on normal distributions. Logistic Regression is a widely used linear classification algorithm that predicts the probability of sample membership in a category. As a simple yet effective model, it performs well in many classification tasks, especially when the relationship between features and the target variable is approximately linear (26).

MLP is a feed-forward neural network used for both classification and regression problems. By learning complex patterns and features through multiple hidden layers (“multilayers”), MLP handles highly nonlinear prediction tasks and finds broad applications in image recognition, natural language processing, and more (27). QDA extends LDA by allowing different covariance matrices for each class, effectively managing cases with complex nonlinear boundaries between categories, particularly suitable for predictions where classes exhibit varying covariance structures (28).

These models have been extensively studied and applied in the field of radiomics, demonstrating their excellent performance and high generalization capabilities (29–31). By utilizing these models for the prediction and classification of radiomics features, we achieved prediction of intrahepatic recurrence in colorectal cancer liver metastasis patients within one year post-surgery, explored the application of integrated models in clinical decision support systems, and aimed to reduce misdiagnosis rates and patient loss through high-precision radiomics grading methods.

### 2.6 Evaluation metrics

This research employs a comprehensive suite of evaluation metrics to ensure objective and thorough assessment of machine learning models, utilizing Accuracy (ACC), Precision (PRE), Recall (REC), and the Receiver Operating Characteristic (ROC) curve with the Area Under the Curve (AUC). These metrics, based on True Positives (TP), True Negatives (TN), False Positives (FP), and False Negatives (FN), offer a multifaceted evaluation of model performance. The formula of ACC (Equation 1), PRE (Equation 2) and REC (Equation 3):

(1) $$ACC=\frac{TP+TN}{TP+FP+TN+FN}$$

(2) $$PRE=\frac{TP}{TP+FP}$$

(3) $$REC=\frac{TP}{TP+FN}$$

## 3 Results

### 3.1 Experimental set-up

To rigorously evaluate the models developed in this study, the dataset was divided into an 80:20 training-to-test ratio for independent testing of model efficacy. During the feature selection phase, to ensure the statistical significance of the features and mitigate computational demands associated with high-dimensionality, a T-test was applied to retain data within a 95% confidence interval. Feature selection was further refined using the Lasso method with an alpha setting of 0.65, aimed at balancing model complexity against performance for optimal feature dimensionality. Additionally, careful adjustments were made to the iterations of various machine learning models to train them to convergence without overfitting, setting each model to run for 50 iterations with a batch size of 512 to ensure comprehensive learning and efficient allocation of computational resources.

All experiments were conducted on a Windows 11 Professional Edition, using Python 3.6.0. The architecture design and validation of models were supported by packages such as Pyradiomics 3.0.1, Scikit-learn 0.24.2, scipy 1.5.4, and matplotlib. The hardware setup included an Intel Core i7 10750H CPU (base frequency 2.6GHz, turbo up to 5GHz, six cores/twelve threads) and an NVIDIA GeForce GTX 4060Ti GPU (16GB memory, 128-bit memory bus).

### 3.2 Results of feature extraction and selection

In this study, we leveraged expert-annotated three-dimensional CT images of patients with colorectal cancer liver metastases to extract radiomic features. Utilizing the Pyradiomics package in Python, we initially extracted 995 radiomic features from the raw CT images. However, due to the large volume and potential irrelevance of many features to the model’s decision-making process, feature selection was imperative to reduce noise and improve model performance.

**Figure 3:**
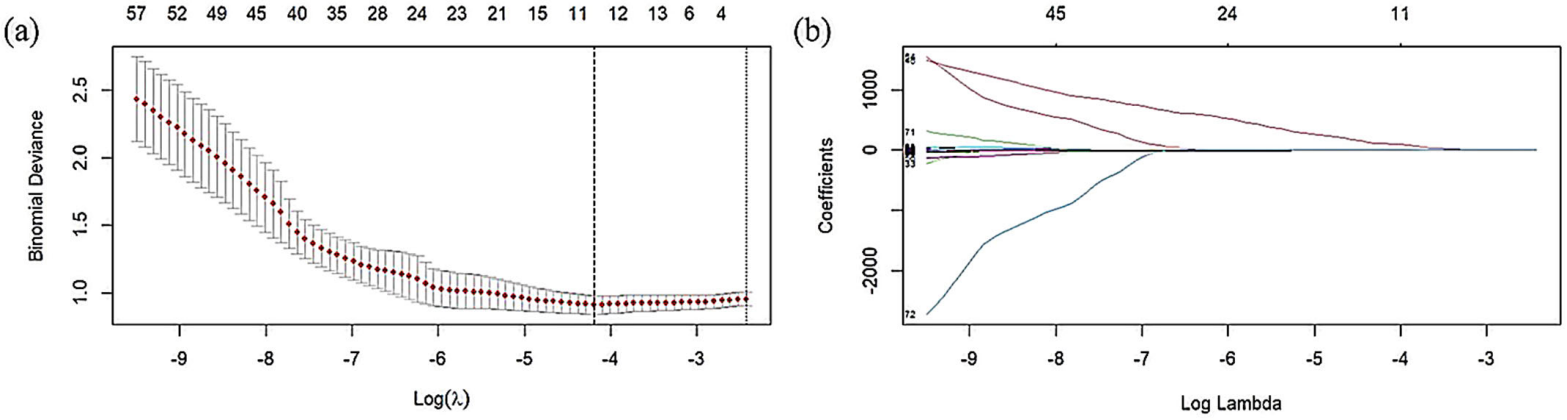
The results of lasso feature selection. **(A)** The cross-validation curve of LASSO. **(B)** The regression coefficient path of LASSO.

Initially, features with a significance level below 0.05 were excluded via T-tests, filtering out features significantly related to the target variable. This T-test yielded 72 features. However, some remaining features irrelevant to the machine learning models could still impact performance. Further feature refinement was conducted using LASSO regression, identifying key predictive features through 10-fold cross-validation. The cross-validation curve and regression coefficient path for LASSO are illustrated in **Figure 3**.

**Figure 4:**
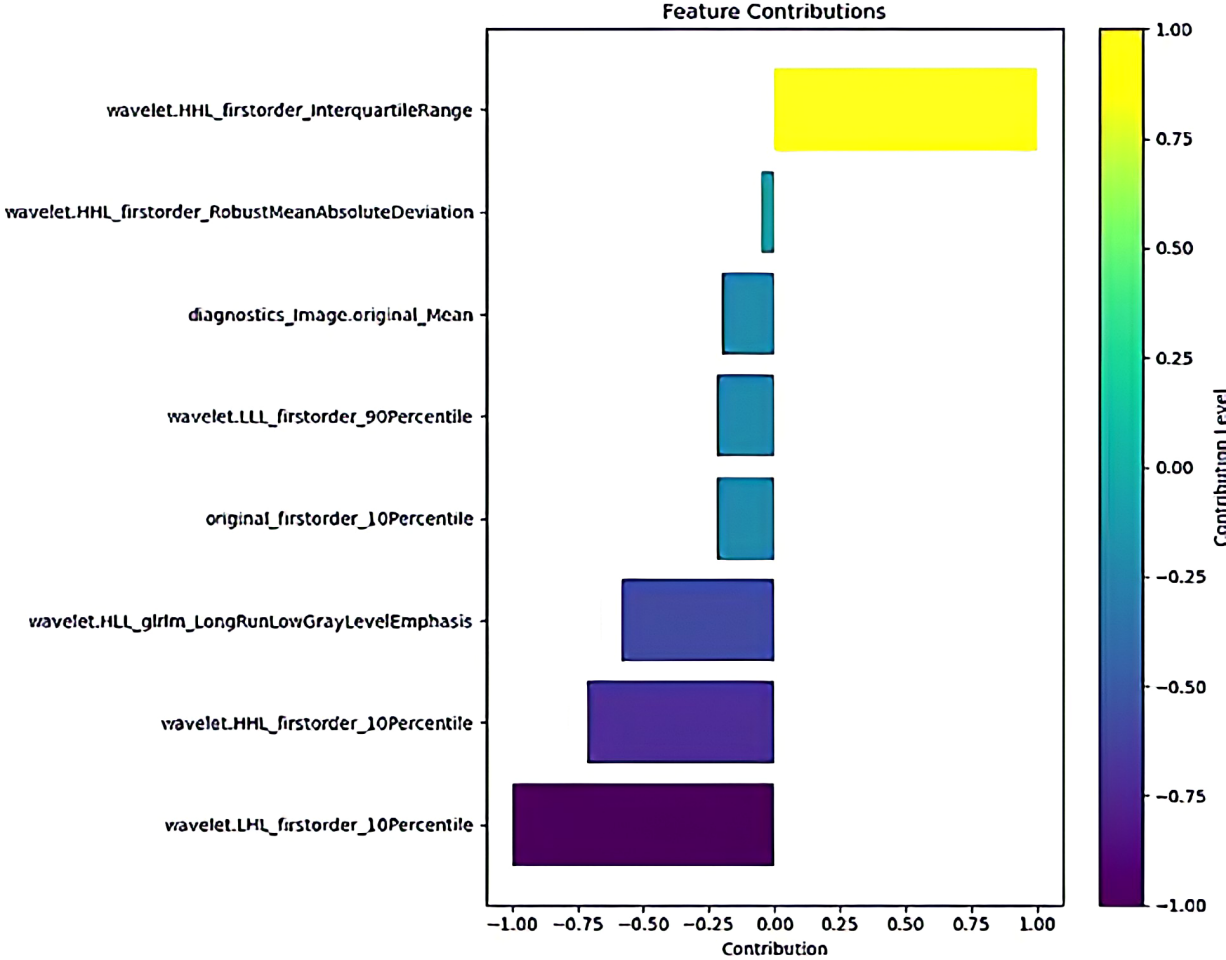
The feature contribution of selected features.

Ultimately, LASSO regression selected 8 essential features, as depicted in the feature contribution curve in **Figure 4**. These features were adequate for training the machine learning model, effectively eliminating the interference from noise and irrelevant features, thus significantly enhancing the model’s performance.

### 3.3 Results of intrahepatic recurrence models

To build more comprehensive models for predicting intrahepatic recurrence within one year post-surgery in patients with colorectal cancer liver metastases, we developed and compared two methodologies: a radiomics-only model and a combined radiomics-clinical model. The radiomics-only model employs radiomic features extracted from CT images, which are input into a machine learning model after feature selection. Conversely, the combined radiomics-clinical model incorporates selected radiomic features with clinical attributes as model inputs. These clinical attributes include patient age, BMI, tumor size, and preoperative chemotherapy status. This integrative approach enables the model to simultaneously consider patient information and clinical indicators of tumor condition, thus enhancing the model’s interpretability and improving its predictive performance.

#### 3.3.1 Results of radiomics models

In this research, features extracted via radiomics were used to train and validate machine learning models for predicting intrahepatic recurrence within one year after surgery in patients with colorectal cancer liver metastases. The performance of these radiomics models is detailed in **Table 2**.

**Table 2:** The result of radiomics models.

Models	ACC	PRE	REC	AUC
AdaBoost	62.07%	84.21%	66.67%	63.23%
Bagging	63.80%	86.49%	66.67%	55.62%
BernoulliNB	17.24%	0	0	50.00%
Calibrated Classifier	65.52%	93.75%	62.50%	71.46%
Decision Tree	63.80%	82.93%	70.83%	50.42%
Extra Tree	62.07%	84.21%	66.67%	56.77%
Gaussian NB	53.45%	92.00%	47.92%	70.83%
Gradient Boosting	72.41%	88.10%	77.08%	66.15%
Kneighbors	55.17%	92.31%	50.00%	60.00%
LightGBM	67.24%	89.19%	68.75%	62.29%
LDA	63.80%	90.91%	62.50%	63.54%
Logistic Regression	50%	82.76%	50.00%	67.50%
MLP	27.59%	100%	12.50%	56.67%
QDA	67.24%	85.37%	72.92%	67.29%
Random Forest	65.52%	83.33%	72.92%	58.33%

**Figure 5:**
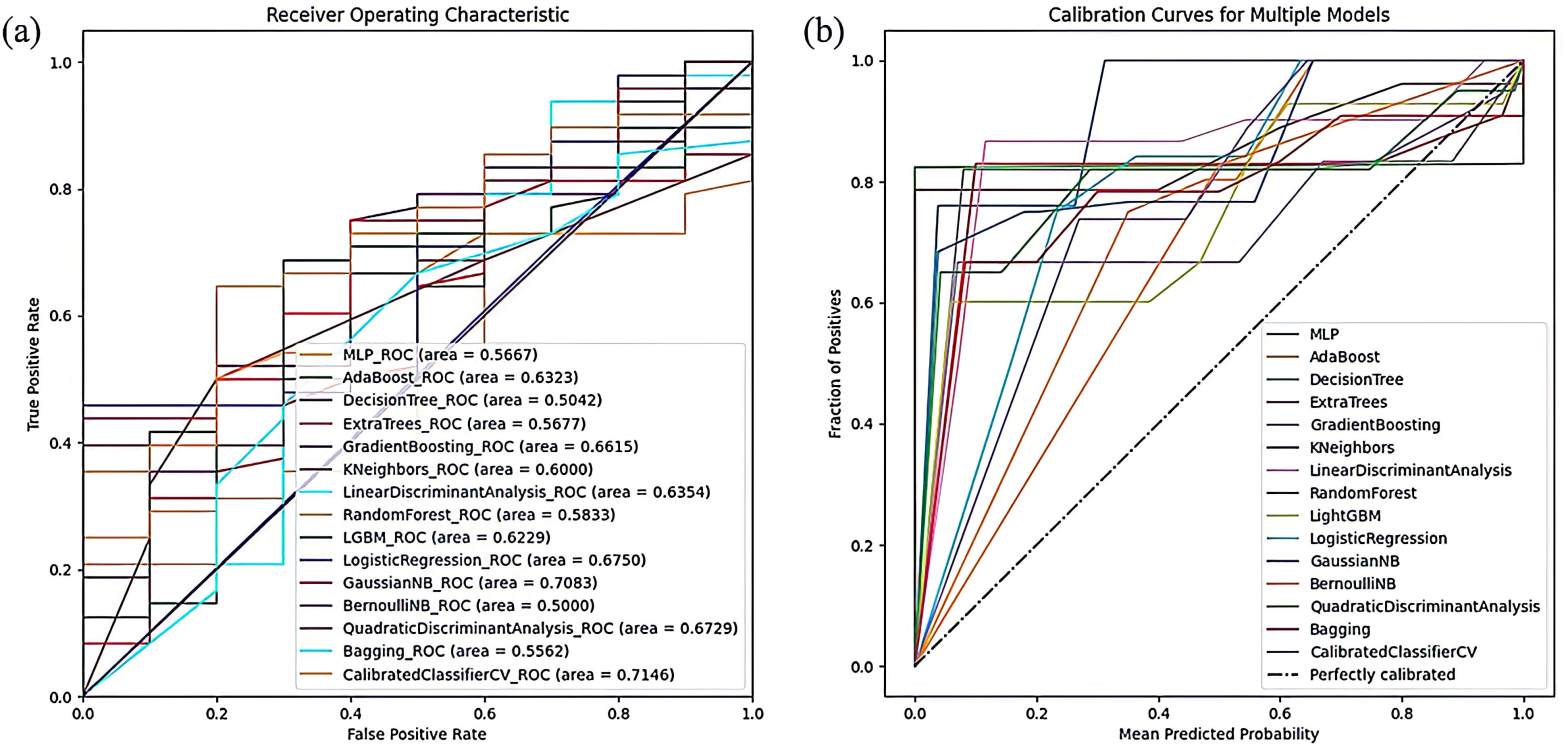
The visualization of model result of radiomics models. **(A)** The ROC curve of radiomics models. **(B)** The Calibration curves of the radiomics models.

The Gradient Boosting classifier showed the highest accuracy among the evaluated models, achieving an average ACC of 72.41% and an AUC of 66.15%. In contrast, the Calibrated Classifier model recorded the lowest misdiagnosis rate, with ACC, PRE, and AUC values of 65.52%, 93.75%, and 71.46%, respectively. The ROC curves and Calibration curves of the radiomics models, depicted in **Figure 5**, reveal that the ROC curve of the Calibrated Classifier model is closest to the upper left corner, while its Calibration curve nears the ideal 45-degree line, demonstrating its outstanding clinical utility.

#### 3.3.2 Results of radiomics-clinical models

To utilize more comprehensive information for training models, we established a radiomics-clinical dataset for training machine learning models. This dataset significantly enhanced model performance compared to using solely radiomic features, as shown in **Table 3**.

**Table 3:** The result of radiomics-clinical models.

Models	ACC	PRE	REC	AUC
AdaBoost	72.41%	86.36%	79.17%	63.54%
Bagging	70.69%	86.05%	77.08%	54.37%
BernoulliNB	70.69%	87.80%	75.00%	65.52%
Calibrated Classifier	56.90%	84.85%	58.33%	66.25%
Decision Tree	67.24%	87.18%	70.83%	60.42%
Extra Tree	74.14%	83.67%	85.42%	67.50%
Gaussian NB	55.17%	89.29%	52.08%	65.62%
Gradient Boosting	75.86%	85.42%	85.42%	63.54%
Kneighbors	56.90%	82.86%	60.42%	45.62%
LightGBM	77.59%	83.02%	91.67%	62.08%
LDA	62.07%	88.24%	62.50%	73.33%
Logistic Regression	51.72%	83.33%	52.08%	61.04%
MLP	44.83%	86.36%	39.58%	63.96%
QDA	82.76%	82.76%	100.00%	49.48%
Random Forest	75.86%	84.00%	87.50%	70.83%

**Figure 6:**
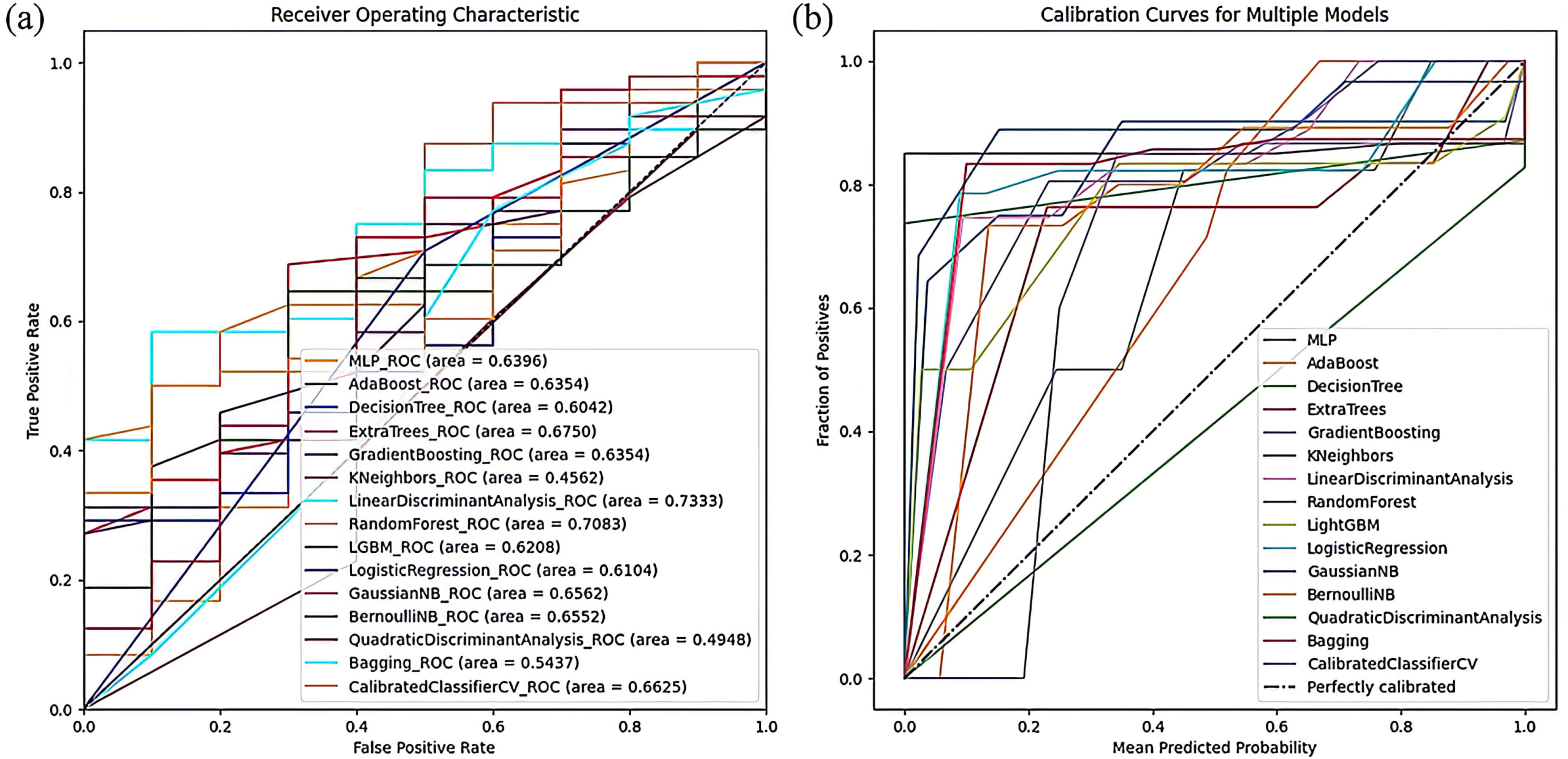
The visualization of model result of radiomics-clinical models. **(A)** The ROC curve of radiomics-clinical models. **(B)** The Calibration curves of the radiomics-clinical models.

In the radiomics-clinical model, the Random Forest classifier exhibited the best performance, achieving an ACC of 75.86% and AUC of 70.83%. It was closely followed by the Gradient Boosting, LightGBM, and Extra Trees models. The ROC curves and Calibration curves of the radiomics-clinical models, displayed in **Figure 6**, show that the ROC curve of the Random Forest model is nearer to the upper left corner and its Calibration curve is closer to the ideal 45-degree line, demonstrating higher reliability and clinical suitability. The use of radiomics-clinical features in the Random Forest model has promising prospects for clinical application, potentially assisting doctors in diagnosis, reducing the workload of radiologists, and minimizing the potential harm caused by diagnostic errors and omissions.

## 4 Discussion

Radiomics facilitates the automatic analysis of numerous image features in a short duration, many of which are difficult to assess visually (32). Integrating radiomics with machine learning models allows for objective and rapid disease classification. This combination provides clinicians with a valuable tool, aiding in the selection of appropriate treatment plans and avoiding unnecessary interventions (33). In this study, the Pyradiomics library was used to extract radiomic data from one-year postoperative CT images of colorectal cancer liver metastasis patients, combined with clinical data, to predict intrahepatic recurrence within one year post-surgery. Fifteen machine learning models were developed and evaluated. The radiomics-clinical model, particularly the Random Forest classifier, demonstrated the highest predictive performance, with an accuracy (ACC) of 75.86% and an area under the curve (AUC) of 70.83%. These promising results have potential clinical applications, helping physicians in diagnosis, reducing the workload of radiologists, identifying features not easily visible to the eye, lowering the rate of misdiagnoses, and ultimately reducing harm to patients.

Colorectal cancer has become the third most common malignant tumor worldwide (1).Liver metastasis is the most common distant metastasis pathway in patients with advanced colorectal cancer. 20% to 25% of patients with colorectal cancer have liver metastases at the time of diagnosis, and up to 50% of patients with colorectal cancer will develop synchronous liver metastases after resection of the primary tumor (34). Surgical radical resection of liver metastases is currently the best way to cure colorectal cancer liver metastases (35).

In clinical practice, some patients with colorectal cancer liver metastases still have a high recurrence rate after surgery, and the benefits of surgery are not obvious. The clinical and pathological characteristics of patients with colorectal cancer liver metastases, including patient characteristics, preoperative treatment, primary tumor and liver metastasis characteristics, and surgical factors, are all related to postoperative recurrence. Currently, the CRS score is the most commonly used evaluation system in clinical practice to predict recurrence and survival in patients with colorectal cancer liver metastases. It is of great value in guiding the timing of surgery and the choice of perioperative treatment, but it is not sufficient to predict the risk of recurrence after resection of liver metastases (36).

Imaging informatics is widely used in the diagnosis, grading and staging, efficacy evaluation and prognosis prediction of tumors by extracting a large number of imaging features from imaging images and analyzing image information in detail (37). This study combined imaging and clinical characteristics to initially establish a visual machine learning prediction model that can predict early intrahepatic recurrence after resection of liver metastases in patients with colorectal cancer liver metastases, thereby providing an effective basis for developing more accurate individualized treatment plans for patients with colorectal cancer liver metastases. For patients in the high-risk group for early recurrence identified by the model, we should take more active measures to check and treat the disease to increase disease control.

This study has several limitations. First, recurrence was evaluated only within a fixed 1-year timeframe; extending follow-up duration and incorporating diverse evaluation criteria would strengthen future analyses. Second, the absence of key biomarkers (RAS/BRAF mutations, MSI status, HER-2 expression) precluded assessment of their prognostic impact. Third, the single-center retrospective design with limited sample size constrains external validity. While genomic integration remains clinically imperative, its implementation requires resource-adaptive methodologies. We propose targeted genetic profiling (e.g., RAS/BRAF PCR) coupled with imaging biomarkers as a pragmatic solution. Our planned pilot study (N = 50-80) will validate this approach using propensity-weighted methods to establish scalable multimodal frameworks.

## 5 Conclusion

CT image omics combined with clinical parameters can predict the risk of early intrahepatic recurrence after surgery in patients with colorectal cancer liver metastases, showing high sensitivity and specificity. It can be used to stratify the risk of recurrence in this group of patients, and more active examination measures and adjuvant treatment can be considered for patients in the high-risk group. In addition, a prospective prediction model combining multiple omics may have higher accuracy, which is also the direction of future research on prediction models.

## Data Availability

The raw data supporting the conclusions of this article will be made available by the authors, without undue reservation.
